# Baseline Oculomotor Parameters Are Prospectively Associated with Cognitive Processing Speed at 6-Year Follow-Up in Multiple Sclerosis: An Exploratory Cohort Study

**DOI:** 10.3390/jcm15103609

**Published:** 2026-05-08

**Authors:** Mariano Ruiz-Ortiz, Cecilia García-Cena, Rosa Hernández-Ramírez, Sara Moreno-García, Andrés Labiano-Fontcuberta, Pablo Montabes-Medina, María del Álamo-Díez, Diego Enrique Guzmán-Villamarín, Julián Benito-León

**Affiliations:** 1 Department of Neurology, 12 de Octubre University Hospital, 28041 Madrid, Spain; samoga1980@gmail.com (S.M.-G.); gandhilabiano@hotmail.com (A.L.-F.); p.montabes1@gmail.com (P.M.-M.); mdelalamodiez@gmail.com (M.d.Á.-D.); 2 Hospital Universitario 12 de Octubre Research Institute (imas12), 28041 Madrid, Spain; 3 Complutense University of Madrid (PhD Program in Medical and Surgical Sciences), 28040 Madrid, Spain; 4 ETSIDI-Center for Automation and Robotics UPM-CSIC, Universidad Politécnica de Madrid, 28012 Madrid, Spain; cecilia.garcia@upm.es; 5 Hospital Universitario de Guadalajara, 19002 Guadalajara, Spain; rosahdez095@gmail.com; 6 Escuela de Ciencias Básicas, Tecnología e Ingeniería, Universidad Nacional Abierta y a Distancia (UNAD), Bogotá 111411, Colombia; 7 Department of Medicine, Faculty of Medicine, Complutense University of Madrid, 28040 Madrid, Spain; 8 Network Center for Biomedical Research in Neurodegenerative Diseases (CIBERNED), 28029 Madrid, Spain

**Keywords:** multiple sclerosis, eye-tracking, smooth pursuit, antisaccade, cognitive processing speed, digital biomarker

## Abstract

**Background/Objectives:** Multiple sclerosis (MS) commonly impairs information processing speed, which is not well captured by conventional disability metrics. Oculomotor paradigms engage neural circuits frequently affected in MS and may provide objective cognitive correlates. We investigated whether baseline oculomotor parameters are prospectively associated with processing speed at ~6-year follow-up. **Methods:** Forty-four patients with MS underwent a standardized oculomotor battery (visually guided saccades, antisaccades, and sinusoidal smooth pursuit). The Symbol Digit Modalities Test (SDMT) was reassessed after a mean of 5.7 years. Age-adjusted Spearman partial correlations were computed between 30 baseline oculomotor parameters and follow-up SDMT, applying false discovery rate (FDR) correction. Sensitivity analyses included Cook’s distance. **Results:** Three parameters survived FDR correction: reflexive saccade duration (i.e., erroneous fixation duration—the dwell time at the erroneous target location before corrective antisaccade initiation; ρ = −0.59, q = 0.0011), catch-up saccades (ρ = −0.52, q = 0.0055), and reflexive saccade latency (ρ = −0.44, q = 0.033). Results remained stable after excluding influential observations. In multivariable analysis (adjusted R^2^ = 0.60), reflexive saccade duration (*p* < 0.001) and catch-up saccades (*p* = 0.019) were independently associated with lower SDMT. **Conclusions:** Baseline antisaccade reflexive saccade duration and smooth-pursuit catch-up saccades were prospectively associated with worse cognitive processing speed at long-term follow-up, suggesting eye-tracking-derived metrics as candidate objective correlates warranting prospective validation in MS.

## 1. Introduction

Cognitive impairment affects 40–70% of patients with multiple sclerosis (MS) and is a leading determinant of unemployment and quality-of-life loss [1,2]. Slowed information processing speed, reliably captured by the Symbol Digit Modalities Test (SDMT), is the most commonly impaired cognitive domain across all disease subtypes and serves as the primary cognitive outcome in MS clinical trials [3,4,5]. Despite its clinical relevance, the early identification of patients destined to show lower processing speed over the following years remains an unmet need: the EDSS captures motor disability poorly sensitive to cognition [6,7], and MRI lesion burden explains only a modest fraction of cognitive variance [8].

Eye-tracking paradigms provide an objective, non-invasive window into the integrity of fronto-parietal and cerebellar circuits that are frequently disrupted in MS and are directly relevant to processing speed [9,10]. Cross-sectional studies have established that antisaccade latency, error rates, and smooth pursuit impairments correlate with cognitive performance in MS [11,12,13]. The antisaccade task requires prefrontal inhibitory control—suppressing the reflexive saccade toward a peripheral stimulus and generating a voluntary movement in the mirror direction—engaging the dorsolateral prefrontal cortex and frontal eye fields [14,15]. Smooth pursuit paradigms, by contrast, engage a cerebello-parieto-frontal predictive system; when the eye falls behind the moving target, compensatory catch-up saccades are generated, quantifying the failure of this predictive mechanism [16,17].

Despite growing cross-sectional evidence, prospective longitudinal studies examining whether oculomotor parameters recorded at a single time point are prospectively associated with cognitive status years later are essentially absent from the MS literature. A systematic scoping review published in 2025 confirmed that virtually all existing evidence is cross-sectional and that no oculomotor parameter has been validated as a longitudinal predictor of cognitive outcomes in MS [16]. Cross-sectional data from the same cohort studied here have previously established group-level oculomotor alterations relative to healthy controls [17], but longitudinal predictive value has not been tested.

The present study addresses this gap using a 6-year prospective follow-up of the same cohort. We examined whether any of the 30 oculomotor parameters derived from a standardized baseline battery were prospectively associated with SDMT score at follow-up, with Spearman partial correlations as the primary analysis—justified by the non-normal distributions of most oculomotor variables—and pre-specified Cook’s distance diagnostics to quantify the influence of individual observations.

## 2. Materials and Methods

### 2.1. Participants and Study Design

This prospective longitudinal cohort study consecutively recruited adult patients with MS from the outpatient neurology clinic of a tertiary referral center (Hospital Universitario 12 de Octubre, Madrid, Spain) between April and May 2018. All participants underwent a standardized oculomotor recording at baseline (2018) and were reassessed clinically and neuropsychologically after a mean follow-up of 5.7 ± 0.5 years (2024). The study was conducted in accordance with the Declaration of Helsinki and received approval from the Ethics Committee of Hospital Universitario 12 de Octubre (approval code 17/035, date of approval: 31 January 2017). Written informed consent was obtained from all participants prior to inclusion.

Eligible participants were aged 18–70 years and fulfilled the 2017 McDonald diagnostic criteria for MS [18]. Best-corrected visual acuity ≥0.8 in at least one eye was required. Exclusion criteria were: neurological conditions other than MS affecting oculomotor or cognitive function; clinically relevant ophthalmological disease (glaucoma, macular degeneration, or diabetic retinopathy); ocular surgery within the preceding six months; and use of medications known to substantially influence eye movements (benzodiazepines or hypnotics) within 24 h before testing. No formal psychiatric exclusion criteria were applied; however, the potential influence of mood and anxiety on oculomotor performance at baseline was not measurable (baseline neuropsychological data were not collected in 2018), and any residual confounding by psychiatric comorbidity cannot be fully excluded. The 24-h benzodiazepine washout applied to acute or as-needed use; patients on long-term benzodiazepine therapy were managed at the examining clinician’s discretion, and the potential influence of chronic use on oculomotor metrics is acknowledged as a limitation.

### 2.2. Clinical Assessment

At both time points, physical disability was rated with the EDSS [6]. At follow-up, cognitive processing speed was assessed using the written SDMT [3]; fatigue impact using the Daily Fatigue Impact Scale (D-FIS) and a 0–100 visual analog fatigue scale [19,20]; anxiety and depression using the Beck Anxiety Inventory (BAI) [21] and the Beck Depression Inventory-II (BDI-II) [22]. Baseline neuropsychological data—including baseline SDMT, fatigue, anxiety, and depression scores—were not collected at the 2018 assessment, constituting an acknowledged limitation of the study design.

### 2.3. Oculomotor Protocol and Data Acquisition

Eye movements were recorded with a non-invasive infrared video-oculography (VOG) system previously validated for accuracy (<0.4°) and precision (<0.035° RMS) in clinical settings [23,24]. Monocular gaze was acquired at 100 Hz using pupil–corneal reflection tracking under near-infrared illumination. Participants were seated 60 cm from a 24-inch monitor in a dimly lit room, with head motion constrained by a chin-and-forehead rest. A nine-point calibration was performed before each test session.

Four paradigms were administered: (i) visually guided saccades in horizontal plane were assessed using step targets presented at ±5°, ±10°, and ±20° (22 repetitions) from which latency, peak velocity, and gain were computed separately for each condition; (ii) the horizontal antisaccade task (step targets at ±5°, ±10°, and ±20°, 22 repetitions), in which participants were instructed to look in the direction opposite to the target, yielding antisaccade latency (Δt, ms), reflexive saccade latency (ms; time from target onset to onset of the involuntary prosaccade toward the target), reflexive saccade duration (Δref, ms; dwell time at the erroneous target location before initiation of the voluntary corrective antisaccade [17]—this parameter quantifies the persistence of the reflexive error before voluntary inhibitory override, and is conceptually distinct from saccade movement duration), error counts, success rates, and correction rates; (iii) horizontal sinusoidal smooth pursuit (stimulus frequency 0.4 Hz, amplitude ±20°, peak target velocity approximately 50°/s, three 8-s trials), from which catch-up saccades (compensatory saccades generated when the eye falls behind stimulus velocity), back-up saccades, pursuit gain, and velocity error were extracted; and (iv) vertical sinusoidal smooth pursuit (identical parameters, three 8-s trials), from which the same variables were derived. A total of 30 oculomotor parameters were computed per participant. Further technical details regarding task design, signal processing, and parameter definitions are provided in the original methodological publication [17]. Clinical records for all 44 included patients were reviewed: no participant had a documented history of acute internuclear ophthalmoplegia (INO) or clinically significant nystagmus on neurological examination at the time of the 2018 recording. Subclinical nystagmus cannot be fully excluded, but no trial was excluded on this basis at quality control.

### 2.4. Statistical Analysis

Statistical analyses were performed in Python 3.12 (NumPy, SciPy, pandas). The Shapiro–Wilk test was used to assess normality. Given that 26 of 30 oculomotor parameters showed significant deviation from normality (Shapiro–Wilk *p* < 0.05), Spearman partial rank correlations—computed as Pearson correlations of age-adjusted rank residuals—were pre-specified as the primary analysis method. Pearson partial correlations, computed on the original scale, are reported as a secondary analysis.

For each of the 30 oculomotor parameters, a Spearman partial correlation with SDMT at follow-up was computed after partialling out baseline age. The Benjamini–Hochberg procedure was applied to control the false discovery rate (FDR) at 5% across the 30 comparisons. For outcomes with FDR-significant predictors, multivariable linear regression models were constructed incorporating independent predictors (selected after collinearity screening using Pearson r; variables with pairwise r ≥ 0.69 were treated as a single cluster, with the variable carrying the clearest biological interpretation retained).

Cook’s distance was pre-specified as a sensitivity analysis to quantify the influence of individual observations on the primary associations. The threshold of 4/n was used to flag highly influential observations. Three analytic scenarios were pre-specified: (1) full sample; (2) excluding observations exceeding Cook’s distance threshold (4/n); and (3) additionally excluding observations flagged by the IQR rule (Q3 + 1.5 × IQR) for the predictor variable. All analyses were complete-case. One participant was missing reflexive saccade duration data (no valid corrective antisaccade trials recorded), and one different participant was missing catch-up saccade data (poor-quality smooth pursuit recording excluded at quality control), each yielding *n* = 42 for the respective analyses; the same 42-patient subset was used for all primary analyses and the combined multivariable model. One participant had a missing SDMT at follow-up and was excluded from all analyses.

## 3. Results

### 3.1. Sample Characteristics

Forty-four patients with MS were included. Demographic and clinical characteristics are summarized in Table 1. At baseline, the predominant disease subtype was relapsing–remitting MS (*n* = 40, 90.9%); median EDSS was 1.5 (IQR 1.0–2.6). Mean disease duration was 11.5 ± 8.8 years and mean age at symptom onset was 31.8 ± 7.7 years. At follow-up (mean 5.7 ± 0.5 years), six patients had converted from RRMS to SPMS during the follow-up period, resulting in seven patients classified as SPMS at follow-up (15.9%), and median EDSS was 2.0 (IQR 1.0–3.6). A comparison of baseline oculomotor parameters between converters (*n* = 6) and non-converters (*n* = 34) was not pre-specified and is not reported; the small cell size would render any such comparison underpowered. Mean SDMT at follow-up was 44.3 ± 9.8 (range 13–66).

### 3.2. Exploratory Screening: Spearman Partial Correlations with SDMT at Follow-Up

**Figure 1:**
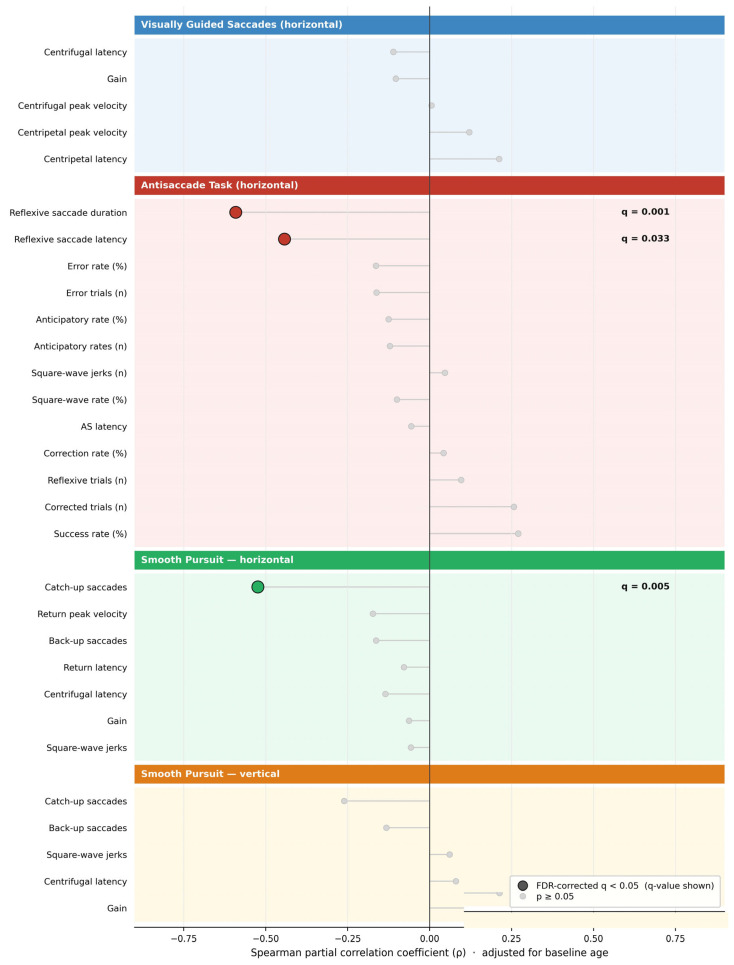
Spearman partial correlation coefficients (adjusted for baseline age) between all 30 baseline oculomotor parameters and SDMT at 6-year follow-up, ranked by absolute effect size. Filled circles with bold stems and q-value annotations indicate the three parameters surviving Benjamini–Hochberg FDR correction (q < 0.05). Circles with lighter stems indicate non-significant associations. All correlations are partial, with baseline age partialled out.

Age-adjusted Spearman partial correlations between all 30 baseline oculomotor parameters and SDMT at follow-up are displayed in Figure 1. Three parameters survived FDR correction (q < 0.05): reflexive saccade duration (ρ = −0.59, *p* < 0.001, q = 0.0011, *n* = 42), catch-up saccades (ρ = −0.52, *p* < 0.001, q = 0.0055, *n* = 42), and reflexive saccade latency (ρ = −0.44, *p* = 0.003, q = 0.033, *n* = 42). No additional parameters reached nominal significance (*p* < 0.05) without also surviving FDR correction. Descriptive statistics for the main oculomotor parameters of interest are provided in Table 2.

### 3.3. Influence Diagnostics

**Figure 2:**
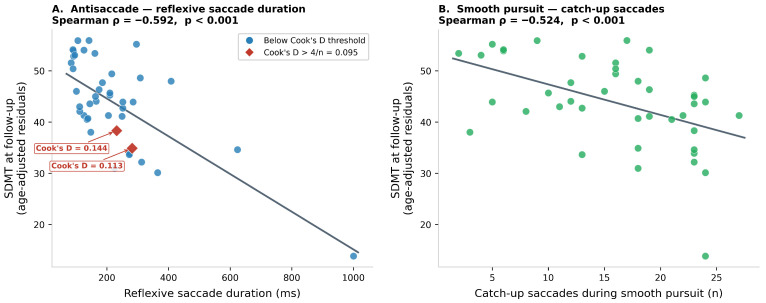
Partial associations between the two main oculomotor predictors and SDMT at 6-year follow-up, after partialling out baseline age from both axes (partial regression display). Each point represents one patient; the regression line (OLS on residuals) is overlaid. (**A**). Antisaccade reflexive saccade duration versus age-adjusted SDMT residuals (Spearman ρ = −0.59, *p* < 0.001). Participants exceeding the Cook’s D threshold (4/*n* = 0.095) are annotated in red. (**B**). Smooth pursuit catch-up saccades versus age-adjusted SDMT residuals (Spearman ρ = −0.52, *p* < 0.001). No Cook’s D outlier was identified for this parameter.

Cook’s distance analysis of the regression of reflexive saccade duration on SDMT (adjusted for age) identified two observations with Cook’s D marginally exceeding the 4/n threshold (Cook’s D = 0.144 and 0.113; threshold = 0.095). Both have Δref values in the intermediate range (232 ms and 282 ms, well within the main distribution) and SDMT values of 30 and 28, respectively. Notably, the participant with the most extreme reflexive saccade duration (1000 ms, SDMT = 13) does not exceed the Cook’s D threshold in the current dataset (Cook’s D = 0.002), as the regression line passes very close to this observation. Two IQR outliers for reflexive saccade duration (>Q3 + 1.5 × IQR = 467 ms) were also identified: reflexive saccade duration = 1000 ms (SDMT = 13) and =624 ms (SDMT = 32). No highly influential observation was identified for catch-up saccades. Partial regression plots for both predictors, with Cook’s D outliers annotated, are displayed in Figure 2.

### 3.4. Sensitivity Analysis: Three Pre-Specified Exclusion Scenarios

**Figure 3:**
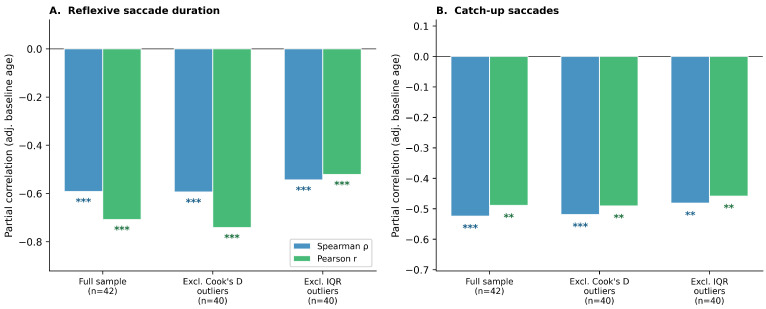
Sensitivity analysis: Spearman partial correlations (blue) and Pearson partial correlations (green) between each oculomotor predictor and SDMT at follow-up across three pre-specified exclusion scenarios: full sample (*n* = 42); excluding Cook’s D outliers (*n* = 40); and excluding IQR outliers for reflexive saccade duration (*n* = 40). Significance markers: *** *p* < 0.001; ** *p* < 0.01. (**A**). Antisaccade reflexive saccade duration. (**B**). Smooth pursuit catch-up saccades.

Results across the three pre-specified scenarios are displayed in Figure 3 and summarized in Table 3. For reflexive saccade duration, both Spearman and Pearson partial correlations were significant across all three scenarios: full sample (ρ = −0.59, r = −0.71), excluding Cook’s D outliers (ρ = −0.59, r = −0.74), and excluding IQR outliers (ρ = −0.54, r = −0.52; all *p* ≤ 0.001). For catch-up saccades, associations were similarly stable: ρ ranged from −0.48 to −0.52 and Pearson r from −0.46 to −0.49 across all three scenarios (all *p* ≤ 0.003). Both associations are therefore robust and independent of the specific observations identified by Cook’s distance or IQR diagnostics.

### 3.5. Multivariable Regression Model

Collinearity screening revealed that reflexive saccade duration, reflexive saccade latency, and antisaccade error metrics were highly intercorrelated (pairwise Pearson r = 0.69–0.99), forming a single antisaccade inhibitory-failure cluster. Reflexive saccade latency was therefore not entered jointly with reflexive saccade duration in the multivariable model; reflexive saccade duration was selected as the single cluster representative for its direct mechanistic interpretability (duration of erroneous fixation before voluntary correction). Catch-up saccades were structurally independent of this cluster (r = 0.21, *p* = 0.18) and entered the model as a distinct predictor. For comparability, the same three exclusion scenarios (full sample, excluding Cook’s D outliers from the reflexive saccade duration model, and excluding IQR outliers for reflexive saccade duration) were applied to catch-up saccades, even though no influential observation was identified for catch-up saccades independently.

In the full-sample multivariable model incorporating age, reflexive saccade duration, and catch-up saccades, adjusted R^2^ was 0.599 (*n* = 42, Table 3). Both oculomotor parameters were independently significant (reflexive saccade duration: β = −0.033, 95% CI [−0.046, −0.021], *p* < 0.001; catch-up saccades: β = −0.348, 95% CI [−0.636, −0.059], *p* = 0.019). Age was not independently significant (*p* = 0.054). When Cook’s D outliers were excluded (*n* = 40), adjusted R^2^ was 0.449, and both predictors remained significant (reflexive saccade duration: β = −0.030, *p* = 0.004; catch-up saccades: β = −0.356, *p* = 0.019). When IQR outliers were excluded (*n* = 40), adjusted R^2^ was 0.425, with both predictors significant (reflexive saccade duration: β = −0.038, *p* = 0.005; catch-up saccades: β = −0.346, *p* = 0.024).

### 3.6. Sensitivity Analysis: Adjustment for Concurrent Fatigue, Anxiety, and Depression

The following sensitivity analysis examines whether the associations of baseline oculomotor parameters with follow-up SDMT are independent of the patient’s concurrent affective and fatigue state at follow-up. Because fatigue, anxiety, and depression were measured concurrently with SDMT at follow-up, they cannot serve as baseline confounders in the primary analysis. To evaluate whether the oculomotor associations were independent of the patient’s affective and fatigue state at the time of follow-up assessment, a pre-specified sensitivity analysis was performed using Spearman partial correlations additionally adjusted for FIS fatigue score, BAI, and BDI-II simultaneously. None of these covariates were independently associated with SDMT (fatigue: *p* = 0.806; BAI: *p* = 0.223; BDI-II: *p* = 0.183; individual Pearson r ranging from −0.13 to −0.07, all *p* > 0.35). The oculomotor associations were virtually unchanged across all tested combinations: reflexive saccade duration (ρ = −0.591, *p* < 0.001, q = 0.0011), catch-up saccades (ρ = −0.481, *p* = 0.001, q = 0.017), and reflexive saccade latency (ρ = −0.471, *p* = 0.002, q = 0.017) all remained FDR-significant. In the extended multivariable regression model including age, FIS, BAI, BDI-II, reflexive saccade duration, and catch-up saccades (*n* = 42, adj-R^2^ = 0.588), only reflexive saccade duration (β = −0.031, *p* < 0.001) and catch-up saccades (β = −0.331, *p* = 0.029) were independently significant, confirming that the oculomotor signal is not explained by concurrent affective or fatigue state.

### 3.7. Physical Disability, Fatigue, Anxiety, and Depression at Follow-Up

In the following analyses, baseline oculomotor parameters were tested for association with follow-up outcomes other than SDMT. No oculomotor parameter survived FDR correction for Expanded Disability Status Scale (EDSS) at follow-up, ΔEDSS, fatigue (D-FIS and visual analog fatigue scale), anxiety, or depression (all q > 0.35). A single nominally significant association was observed for EDSS: horizontal smooth pursuit velocity error (ρ = 0.41, *p* = 0.006, q = 0.35, adjusted for age and baseline EDSS), which did not survive FDR correction. The absence of 2018 baseline measures for these outcomes further limits inference, as change scores could not be calculated.

## 4. Discussion

The main finding of this prospective 6-year study is that two oculomotor parameters recorded at baseline were independently associated with cognitive processing speed at follow-up in MS: the duration of reflexive saccades in the antisaccade task and catch-up saccades during horizontal sinusoidal smooth pursuit. Both parameters survived FDR correction, remained independently associated in a joint multivariable model (adjusted R^2^ = 0.60), and were robust across pre-specified Cook’s distance, IQR, and concurrent-covariate sensitivity analyses. Baseline EDSS, by contrast, was not associated with SDMT at follow-up (r = −0.21, *p* = 0.17), confirming that oculomotor parameters capture information not encoded in conventional disability metrics.

Smooth pursuit catch-up saccades are generated when the cerebello-parieto-frontal predictive system fails to maintain the velocity of a moving target, requiring corrective saccadic jumps to prevent excessive retinal slip [25,26]. Their count during sinusoidal smooth pursuit has previously been shown to be elevated in MS relative to healthy controls [17], and cross-sectional associations with EDSS have been reported inconsistently [17,26]. The present findings extend this picture longitudinally: the count of catch-up saccades at baseline was associated with—independently of age—lower cognitive processing speed six years later. The biological link may reflect that catch-up saccades index the failure of cerebellar predictive coding, and that cerebellar contributions to cognition, particularly to processing speed and temporal prediction, are increasingly recognized as relevant in MS [27,28,29]. As reported in the Results, baseline EDSS was not associated with follow-up SDMT (unadjusted Spearman ρ = −0.21, *p* = 0.17), confirming that oculomotor parameters capture information not encoded in conventional disability metrics.

The antisaccade reflexive saccade duration quantifies how long the eye dwells at the erroneous target location before the voluntary corrective movement is initiated—a direct index of the temporal depth of prefrontal inhibitory failure [14,15,24]. Its association with follow-up SDMT in rank-based analyses is biologically plausible: both parameters reflect processing efficiency within fronto-parietal networks commonly disrupted in MS. The sensitivity analysis further strengthens confidence in this association. First, when the two IQR outliers for reflexive saccade duration are excluded—the participant with the longest reflexive saccade duration (1000 ms, SDMT = 13) and the second extreme case (624 ms, SDMT = 32)—the Spearman correlation remains ρ = −0.54 (*p* = 0.0003) and the Pearson r = −0.52 (*p* = 0.0006). Cook’s distance analysis reveals that these extreme observations do not, in fact, drive the association: the participant with Δref = 1000 ms has Cook’s D = 0.002 (far below the threshold of 0.095), because their observed SDMT (13) falls nearly on the regression line. The two observations that marginally exceed the Cook’s D threshold (indices 10 and 16) have intermediate Δref values (232 and 282 ms) and do not correspond to obvious outliers—their leverage arises from combinations of moderate residuals and somewhat elevated hat values. Further, the association was maintained when additionally adjusting for concurrent fatigue, anxiety, and depression at follow-up (all of which were negligibly associated with SDMT), confirming that the oculomotor signal is not a proxy for the patient’s affective state at the time of assessment.

Several important limitations must be acknowledged. The sample size (*n* = 40–42 per analysis) is small for a longitudinal study with 30 candidate parameters, limiting statistical power and increasing the risk of inflated effect size estimates. The explained variance in the full multivariable model (adj-R^2^ = 0.60) should be treated as an optimistic, potentially inflated estimate: the predictors were selected by screening 30 variables without formal penalisation or cross-validation, and no internal or external validation was performed. Effect size estimates are therefore liable to winner’s curse inflation and may not replicate at their observed magnitude in an independent sample. Critically, SDMT was not measured at baseline, precluding calculation of a change score; the SDMT at follow-up reflects the cumulative cognitive trajectory including unknown baseline status. Because patients with lower baseline cognitive reserve might show lower absolute SDMT at follow-up independently of any oculomotor-mediated process, the observed associations cannot be equated with prediction of cognitive deterioration or decline. The cohort is single-center, predominantly relapsing–remitting, and drawn from a tertiary referral center, limiting generalizability. Finally, 30 simultaneous comparisons require FDR correction and cautious interpretation of nominally significant results.

Notwithstanding these limitations, this appears to be one of the first studies to demonstrate a longitudinal association between oculomotor parameters and cognitive processing speed in MS over a multi-year horizon. Both oculomotor parameters are measurable non-invasively within a standard clinical visit, supporting their evaluation as candidate objective correlates of cognitive processing speed in MS [30]. These findings are best viewed as hypothesis-generating: without baseline cognitive data, the associations cannot be equated with prediction of cognitive decline, and replication in larger, independent cohorts with baseline SDMT is required before translational claims can be substantiated. If confirmed, oculomotor screening might assist in identifying patients who warrant closer neuropsychological monitoring; however, clinical application would require demonstration of incremental predictive validity over established markers in a prospectively designed, powered study.

## Figures and Tables

**Table 1:** Demographic and clinical characteristics of the study cohort (*n* = 44).

Characteristic	Value
**Demographics**	
Age at baseline (years), mean ± SD	43.2 ± 9.9
Sex, female, n (%)	27 (61.4%)
Age at symptom onset (years), mean ± SD	31.8 ± 7.7
Disease duration at baseline (years), mean ± SD	11.5 ± 8.8
Follow-up duration (years), mean ± SD	5.7 ± 0.5
**MS subtype at baseline, n (%)**	
Relapsing–remitting	40 (90.9%)
Secondary progressive	1 (2.3%)
Primary progressive	2 (4.5%)
Clinically isolated syndrome	1 (2.3%)
**MS subtype at follow-up, n (%)**	
Relapsing–remitting	34 (77.3%)
Secondary progressive	7 (15.9%)
Primary progressive	2 (4.5%)
Clinically isolated syndrome	1 (2.3%)
**Disability**	
EDSS at baseline, median (IQR)	1.5 (1.0–2.6)
EDSS at follow-up, median (IQR)	2.0 (1.0–3.6)
ΔEDSS (follow-up − baseline), mean ± SD	0.68 ± 1.28
Worsened (ΔEDSS > 0), n (%)	22 (50.0%)
Stable (ΔEDSS = 0), n (%)	17 (38.6%)
Improved (ΔEDSS < 0), n (%)	5 (11.4%)
**MRI and lesion activity**	
T2 lesion load < 10, n (%)	5 (11.4%)
T2 lesion load 10–50, n (%)	29 (65.9%)
T2 lesion load > 50, n (%)	10 (22.7%)
New T2 lesions 2018–2024, median (IQR)	0.5 (0–3.0)
**Follow-up outcomes**	
SDMT, mean ± SD (range)	44.3 ± 9.8 (13–66)
Fatigue (D-FIS, 0–32), mean ± SD	12.0 ± 8.8
Fatigue VAS (0–100), mean ± SD	48.2 ± 28.3
Anxiety (BAI), mean ± SD	15.2 ± 15.0
Depression (BDI-II), mean ± SD	12.9 ± 9.7

BAI = Beck Anxiety Inventory; BDI-II = Beck Depression Inventory-II; EDSS = Expanded Disability Status Scale; D-FIS = Daily Fatigue Impact Scale; IQR = interquartile range; SDMT = Symbol Digit Modalities Test; VAS = visual analog scale.

**Table 2:** Descriptive statistics of the oculomotor parameters of primary interest.

Parameter	Task	n	Mean ± SD	Median (IQR)	SW *p*
Reflexive saccade duration (ms)	Antisaccade	42	209 ± 160	166 (112–232)	<0.001
Reflexive saccade latency (ms)	Antisaccade	42	530 ± 242	476 (401–551)	<0.001
Error count (n)	Antisaccade	42	0.4 ± 1.3	0 (0–0)	<0.001
Error rate (%)	Antisaccade	42	3.5 ± 11.1	0 (0–0)	<0.001
Catch-up saccades (n)	Smooth pursuit H	42	17.3 ± 11.2	18 (12–23)	<0.001

SW *p* = Shapiro–Wilk normality test *p*-value. H = horizontal.

**Table 3:** Sensitivity analysis: Spearman and Pearson partial correlations (adjusted for baseline age) for the two main oculomotor predictors across three pre-specified exclusion scenarios, and multivariable regression models.

Scenario	n	Spearman ρ	*p* (ρ)	Pearson r	*p* (r)	Multivariable Model (Age + RSD + Catch-Up)
***A. Reflexive saccade duration (antisaccade task) → SDMT***						
Full sample	42	−0.59	<0.001	−0.71	<0.001	adj-R^2^ = 0.599; RSD β = −0.033 (*p* < 0.001 ***); catch-up β = −0.348 (*p* = 0.019 *)
Excl. Cook’s D outliers (Cook’s D outliers)	40	−0.59	<0.001	−0.74	<0.001	adj-R^2^ = 0.449; RSD β = −0.030 (*p* = 0.004 **); catch-up β = −0.356 (*p* = 0.019 *)
Excl. IQR outliers (IQR outliers)	40	−0.54	<0.001	−0.52	0.001	adj-R^2^ = 0.425; RSD β = −0.038 (*p* = 0.005 **); catch-up β = −0.346 (*p* = 0.024 *)
***B. Catch-up saccades (horizontal smooth pursuit) → SDMT***						
Full sample	42	−0.52	<0.001	−0.49	0.001	(see above)
Excl. Cook’s D outliers (Cook’s D outliers)	40	−0.52	0.001	−0.49	0.001	—
Excl. IQR outliers (IQR outliers)	40	−0.48	0.002	−0.46	0.003	—

RSD = reflexive saccade duration. Spearman partial correlations (rank-based) are the pre-specified primary analysis. Pearson partial correlations are secondary. Cook’s D outliers = participants with Cook’s D > 4/*n* = 0.095 threshold (Cook’s D = 0.144 and 0.113; both with intermediate reflexive saccade duration values). IQR outliers = two participants with reflexive saccade duration > Q3 + 1.5 × IQR = 467 ms (values: 1000 ms, SDMT = 13; and 624 ms, SDMT = 32). Multivariable model combines age, RSD, and catch-up saccades. * *p* < 0.05; ** *p* < 0.01; *** *p* < 0.001.

## Data Availability

The data that support the findings of this study are not publicly available due to patient privacy constraints. De-identified data may be made available to qualified researchers upon reasonable request to the corresponding author.
